# Social media use for healthcare professionals in South Africa: The do’s and don’ts

**DOI:** 10.4102/safp.v68i1.6319

**Published:** 2026-06-18

**Authors:** Mareike Rabe

**Affiliations:** 1 Vita Oncology, Cape Gate and Bellville, Cape Town, South Africa

**Keywords:** social media, healthcare professionals, ethics, law, professional responsibilities

## Abstract

Social media has become a dominant mode of communication, education and advocacy in healthcare, offering healthcare professionals (HCPs) opportunities to connect with peers, patients and the public. However, its use carries significant ethical, legal and professional responsibilities. In South Africa, the Health Professions Council of South Africa (HPCSA) has responded to these challenges with updated guidance in Booklet 16: Ethical Guidelines on Social Media. This CPD article synthesises HPCSA guidance and complementary resources into a practical framework for responsible social media use by HCPs. The proposed framework: ‘Protect Patients, Protect Yourself, Protect the Profession’, offers actionable recommendations to ensure that online conduct upholds patient rights, safeguards professional integrity and maintains public trust in healthcare. By integrating HPCSA guidelines with international resources such as the World Medical Association’s statement on social media and secure communication platforms such as Celo and Hucu, this article provides HCPs with a structured approach to navigating the complexities of digital professionalism.

## Introduction

Over the last two decades, social media has become an integral part of modern communication, content creation and consumerism, offering healthcare professionals (HCPs) opportunities to learn, educate, advocate and connect with peers, students, mentors, teachers, colleagues, patients and the public.^1,2,3,4^ Yet, its use carries legal, ethical and professional responsibilities. In South Africa (SA), the Health Professions Council of South Africa (HPCSA) has issued clear guidance on responsible online conduct, most notably in Booklet 16: Ethical Guidelines on Social Media, which was updated in March 2025 (hereafter referred to as Booklet 16).^5^

Social media describes ‘any online tools and electronic communication platforms that are used for social networking, sharing content such as opinions, information, photos, videos and audio’.^5^ These may include, but are not limited to, social networks (e.g. Facebook, LinkedIn), content-sharing platforms (e.g. WhatsApp, Instagram, YouTube, X, TikTok), personal and professional blogs, Internet discussion forums and the comment sections of websites.^5,6,7^

As at October 2025, SA had 29.1 million social media user identities, representing 44.9% of the population and reflecting a year-on-year increase of 3.8 million (15.2%). Globally, 5.66 billion social media user identities were recorded.^8^

While individuals typically make medical decisions in accordance with their own views and values, these are often shaped or influenced by their experiences, the environment and their social circle, whether in-person or online.^9^ In the healthcare context, patients do not necessarily use social media to circumvent HCPs, but rather as a complement to HCP services to fulfil the patients’ needs that the HCP cannot meet.^10^

This CPD article aims to synthesise the HPCSA guidelines and complementary resources on social media use into practical recommendations for HCPs.

## Legal considerations

Booklet 16 speaks to HCPs’ obligations on the *Ethical Rules of Conduct for Health Practitioners Registered under the Health Professions Act 1974*, as amended^11^ and other pieces of relevant legislation, such as the *Promotion of Access to Information Act* (*PAIA*),^12^ and the *Protection of Personal Information Act* (*POPIA*).^13^ Table 1 summarises these frameworks.

**TABLE 1:** Social media conduct in healthcare: Practical examples of legal and ethical compliance.

Theme	Resources	Examples of appropriate conduct	Examples of inappropriate conduct
Confidentiality and privacy	HPCSA Booklet 5 (Confidentiality)^21^ *POPIA*^13^ *Health Professions Act*^11^ *Bill of Rights*^14^	A doctor posts a de-identified photo of a wound for educational discussion in a closed professional group, noting that consent was obtained. A clinic uses an encrypted messaging app to share lab results with patients who have opted in. A practitioner reminds colleagues in a WhatsApp group not to include patient names.	A nurse shares a selfie with a patient visible in the background on Instagram without consent. A doctor discusses a celebrity’s diagnosis on LinkedIn without consent. A receptionist posts screenshots of appointment lists on Facebook to show how busy the practice is.
Informed consent and autonomy	HPCSA Booklet 9 (Informed Consent)^22^ HPCSA Booklet 1 (General Ethics)^20^ *Bill of Rights*^14^ WMA Statement on Social Media^26^	Before posting a success story on their social media sites, a physiotherapist asks the patient for written consent and explains how the post will be used. A psychologist clarifies that online testimonials are voluntary and may be withdrawn. A GP uses anonymised case examples in a live YouTube webinar after confirming consent.	A surgeon posts ‘before-and-after’ photos on the practice social media platforms without consent. A GP forwards patients’ comments from WhatsApp to a specialist without consent. A practitioner assumes consent because the patient follows their Facebook page.
Professional conduct and boundaries	HPCSA Booklet 1 (General Ethics)^20^ HPCSA Booklet 16 (Social Media)^5^ *Health Professions Act^11^* WMA Statement on Social Media.^26^	A family physician shares verified health tips from the Department of Health on TikTok and corrects misinformation. A specialist keeps separate personal and professional WhatsApp accounts. A doctor declines to give medical advice in comment threads, referring users to proper consultations. A physician adds a disclaimer to their social media platforms: ‘This is general information, not personal medical advice’.	A practitioner argues with patients in public comment sections on Facebook. A clinician advertises ‘miracle cures’ or exaggerates qualifications on their social media platforms. A doctor posts memes mocking patients or colleagues on LinkedIn.
Telehealth and digital care	HPCSA Booklet 10 (Telehealth)^23^ *POPIA*^13^ WMA Statement on Social Media^26^	A psychiatrist conducts video consultations via a secure telehealth platform and documents consent for virtual care. A GP explains to patients that WhatsApp is used only for appointment scheduling and reminders, not clinical advice. A physiotherapist ensures all telehealth notes are stored on encrypted platforms and systems.	A doctor gives diagnostic advice through Facebook Messenger. A nurse sends lab results via personal email. A practitioner livestreams parts of a teleconsultation for educational purposes without consent.
Access to information and transparency	*PAIA*^12^ *Bill of Rights*^14^ WMA Statement on Social Media^26^	A public health officer posts verified outbreak updates from NICD sources on their social media platforms. A hospital shares its visiting-hours policy on social media to improve access. A clinician explains in an electronic newsletter posted on their social media platforms how patients can request their records under PAIA.	A staff member leaks internal memos online. A practitioner shares confidential internal disciplinary outcomes to a friend via direct messaging. A clinic refuses to correct misinformation about the services it can provide on its social media platforms.
Media appearances and public messaging	WMA Guidelines on Promotional Mass Media Appearances^27^	A physician appears on a social media-streamed podcast to explain vaccination benefits, citing peer-reviewed evidence. A doctor discusses the risks and side effects of a new therapy on his YouTube channel.	A physician endorses a specific brand of supplements on a podcast streamed on social media platforms. A clinician promotes unproven treatments in an interview streamed on social media platforms.

Note: Please see the full reference list of. Rabe M. Social media use for healthcare professionals in South Africa: The do’s and don’ts. S Afr Fam Pract. 2026;68(1), a6319. https://doi.org/10.4102/safp.v68i1.6319.

HPCSA, Health Professions Council of South Africa; POPIA, *Protection of Personal Information Act*; PAIA, *Promotion of Access to Information Act*; NICD, National Institute of Communicable Diseases; WMA, World Medical Association; GP, general practitioner.

According to the *Bill of Rights of the Constitution of the Republic of SA*, every person has the right to approach the court to enforce their rights when they are threatened or violated.^14^ These include, but are not limited to: (1) have dignity protected and respected, (2) physical and psychological integrity, (3) equality, (4) privacy and (5) freedom of expression. Many of the rights are not absolute, but their infringement ought to be justifiable and reasonable in an open and democratic society.^7,14^

As it relates to social media, the first case in SA where damages were awarded to the plaintiff for a defamatory statement made on Facebook transpired in 2013.^7,15^ Further cases have been taken to court after this ruling. In the healthcare context, the HPCSA recently found a HCP guilty of unprofessional conduct and imposed a fine of R10 000.00 for social media posts deemed abusive and damaging to the profession’s reputation. The ruling, based on X posts made in 2025 (which have since been deleted), sparked global debate regarding free speech versus ethical standards.^16,17^ These cases may demonstrate the ‘disinhibition effect’, defined as the lowering in the online social environment of the psychological restraints that normally serve to regulate behaviour.^7,18,19^ In both cases, individuals may have expressed themselves more freely and impulsively online than they would in face-to-face interactions. The relative anonymity, perceived distance and informal nature of social media platforms reduce self-restraint, leading to statements that may breach legal, ethical or professional boundaries.^7,18,19^ These cases highlight how lowered inhibitions while on social media can result in possible reputational harm, legal liability and professional sanctions, underscoring the tension between freedom of expression and the ethical obligations of HCPs.

## Ethical considerations

There are ethical obligations and responsibilities imposed on HCPs regarding their relationships with their patients and each other, such as those set out in the General Ethical Guidelines for Health Care Professionals (Booklet 1)^20^ and Confidentiality: Protecting and Providing Information (Booklet 5).^21^ Obligations relating to the electronic storage and transmission of health records for professional purposes are found in General Ethical Guidelines for Management of Health Records (Booklet 9)^22^ and Ethical Guidelines for Good Practice in Telehealth practice (Booklet 10).^23^

Pontarelli et al.^9^ explored the frequency of ethical transgressions sanctioned by the HPCSA in the context of increased patient empowerment, as a result of the accelerated use of social media by patients between 2014 and 2023. Of the 1012 ethical transgressions among 452 registered HCPs, fraudulent conduct, including falsifying status or qualifications, false advertising material, fraudulent medical records and financial reports and fraudulent billing constituted the predominant complaints. Between 2006 and 2016, there was a reported 100% increase in the number of complaints submitted to the HPCSA.^9^ This increase in lodged complaints has been attributed to the HPCSA distributing pamphlets directing patients on how to place complaints, as well as an increase in patients’ awareness of their rights. This increase may also be likely because of the increasing use of social media in the modern age and will continue to grow as more people flock to various platforms to gather information and communicate.^9^ The study identified the need for an HCP ethics education plan that offers more than a mere check-a-box exercise and recommended an education programme like that of the Minnesota Board of Dentistry and Dr. Bebeau^24,25^ that would foster individualised reflection on ethical shortcomings, strengthen SA’s healthcare sector and build public trust by cultivating strong patient-professional relationships.^9^

The World Medical Association’s Statement on Social Media^26^ and Guidelines on Promotional Mass Media Appearances^27^ underscore that HCPs’ presence in social and mass media must uphold the same ethical standards as clinical practice. Online communication should protect patient confidentiality, respect professional boundaries and provide accurate, evidence-based information. Physicians are cautioned against using social media or promotional appearances to advertise products, endorse unproven treatments or exaggerate qualifications. Instead, their digital and media engagement should serve public health education, transparency and trust, ensuring that professionalism and integrity remain paramount over visibility or commercial gain.

## How should healthcare professionals interact with social media?

Based on the previous sections, the framework of ‘Protect Patients, Protect Yourself, and Protect the Profession’ is presented as a guide for HCPs, with Table 1 illustrating practical examples of recommended do’s and don’ts.^2,5,6,7,9,11,12,13,14,21,28^

### Protect patients

Patients have an enduring right to privacy and confidentiality, with few exceptions. Before sharing any personal health information, especially online, HCPs must obtain written informed consent and remove all patient-identifiable details. If disclosure is necessary, it should be limited to the minimum required, with HCPs full awareness that once published, content may be redistributed beyond its original context. This duty of confidentiality remains in effect even after a patient’s death.

The *POPIA* governs how personal data are processed in SA, including on social media platforms. Healthcare professionals must ensure compliance when handling patient information digitally.

Social media can erode the boundaries of the practitioner–patient relationship. Healthcare professionals should be cautious when approached by patients outside formal healthcare settings and consider the ethical implications before engaging. Inappropriate contact should be met with a respectful reassertion of professional boundaries. Except in emergencies, clinical advice should not be offered via social media. Any health-related content shared online must be generic, evidence-based and accompanied by a clear recommendation to seek in-person consultation should healthcare concerns arise.

### Protect yourself

Healthcare professionals should adopt strong privacy and security practices when using social media. Even with optimised privacy settings, complete anonymity is not assured. Posts may be copied, shared or stored indefinitely, even after deletion. Once content is online, it may persist in caches or be reproduced by others. Secure digital communication platforms, such as Celo^29^ and Hucu,^30^ are secure digital platforms for communication, collaboration and data storage.

To safeguard personal and professional integrity, practitioners should avoid using social media when tired, emotionally distressed or under the influence of alcohol.

### Protect the profession

A HCP’s online presence reflects not only on their individual professionalism but also on the broader reputation of the healthcare profession. Social media conduct must uphold the dignity of clinical practice. Healthcare professionals should refrain from posting defamatory remarks, engaging in hate speech or violating copyright. Concerns about a colleague’s competence or reputation should be addressed privately and through appropriate professional channels, rather than in public forums.

Traditional boundaries between teachers and trainees apply equally in digital spaces. Online interactions should respect and maintain professional roles. For example, a senior clinician may redirect a student’s social media request to formal channels such as email or institutional platforms, ensuring that the relationship remains professional and aligned with ethical standards. Including disclaimers on personal profiles (clarifying that views expressed are personal and not representative of the profession or institution) is recommended.

Healthcare professionals should avoid endorsing hospitals, products, or services in ways that might create a conflict of interest. The HPCSA regulations prohibit canvassing (the promotion of one’s professional goods and services by drawing attention to one’s personal qualities, such as superior knowledge, quality of service, professional guarantees or best practice) and touting (drawing attention to one’s professional goods or services by offering guarantees or benefits that fall outside one’s scope of practice).

### A word on WhatsApp

While not covered in detail in this article, HCPs are strongly encouraged to consult the *Guidelines for the Use of WhatsApp Groups in Clinical Settings in South Africa*^31^ for further direction and recommendations on the use of this platform in their everyday practice.

## Conclusion

Social media offers powerful tools for communication and advocacy in family practice. However, its responsible use requires adherence to ethical and legal frameworks and ongoing professional reflection. By following HPCSA and other guidelines and applying practical do’s and don’ts, HCPs can harness social media to strengthen patient trust, advance public health and uphold the integrity of the profession.
